# Potassium channels in the sinoatrial node and their role in heart rate control

**DOI:** 10.1080/19336950.2018.1532255

**Published:** 2018-10-09

**Authors:** Qadeer Aziz, Yiwen Li, Andrew Tinker

**Affiliations:** William Harvey Heart Centre, Barts & The London School of Medicine & Dentistry, Queen Mary, University of London, London, UK

**Keywords:** ATP-sensitive potassium channel, heart rate, pacemaking, potassium channel, sinoatrial node

## Abstract

Potassium currents determine the resting membrane potential and govern repolarisation in cardiac myocytes. Here, we review the various currents in the sinoatrial node focussing on their molecular and cellular properties and their role in pacemaking and heart rate control. We also describe how our recent finding of a novel ATP-sensitive potassium channel population in these cells fits into this picture.

## Introduction

The regular beating of the heart is generated by intrinsic pacemaker activity in the sinoatrial node (SAN). Heart rate can be easily measured and is known to be associated with disease in a number of epidemiological studies. For example, in Paris Prospective Study I the risk of sudden death increased over three fold (relative risk 3.9) in those with a resting heart rate >75 beats per minute compared to those with one under 60 [1]. Furthermore, the increase in heart rate during exercise and the rate of recovery of rate after cessation of exercise were also strongly associated with sudden death [1]. Moreover, direct intervention to lower heart rate using drugs with predominant effects on the SAN can improve outcomes in heart failure [2].

**Figure 1.**
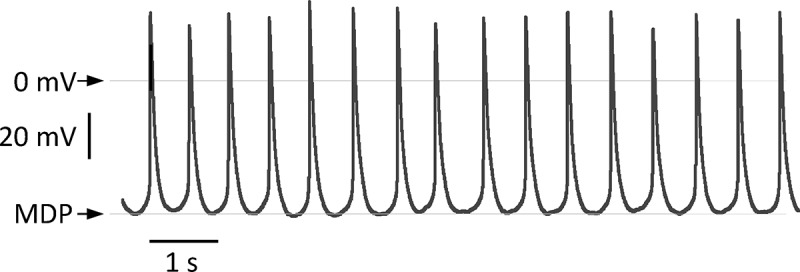
The figure shows a recording of spontaneous action potentials in a single isolated SAN myocyte recorded in current clamp in the whole cell configuration of the patch clamp. It illustrates the characteristic morphology of the action potential. The line indicates 0 millivolt and the maximum diastolic potential was −50 mV.

The pacemaker action potential has a characteristic morphology with a diastolic depolarization before a threshold is reached with a consequent action potential. Figure 1 shows this in single mouse SAN cells studied in the current clamp mode of the patch clamp. Exactly how the intrinsic pacemaker clock is generated is controversial. Historically the rhythmical oscillations of membrane potential were thought to be governed by the hyperpolarisation-activated cyclic nucleotide-gated cation channel (also known as the “funny” current, I_f_) constituted of the product of the HCN genes (largely HCN4) [3]. However, published data now show the importance of intracellular calcium cycling specifically diastolic calcium release from the sarcoplasmic reticulum generating an inward current via the sodium-calcium exchanger [4]. It is likely that these two mechanisms are in fact highly interdependent. Furthermore, this oscillatory electrical activity is subject to regulation by the autonomic nervous system and that allows heart rate to increase flexibly with increased demand such as during exercise. In this review, we consider the role of potassium channels in shaping SAN electrophysiology and their part in heart rate control. We also discuss our recent work on a new player in SAN excitability namely ATP-sensitive potassium channels (K_ATP_) constituted of Kir6.1 subunits.

## An overview of the electrophysiology of the SAN

The SAN sits in the upper part of the right atrium laterally to the entrance of the superior vena cava. Textbooks show a characteristic pacemaker action potential, however the SAN is actually quite a heterogeneous structure, both morphologically and electrophysiologically [5]. The primary pacemaker can also shift location with changes in autonomic tone [6]. With this caveat in mind it is helpful to consider the main currents underlying excitability.

The rapid initial depolarization of the action potential, also known as phase 0, is not as fast as in ventricular tissue and is mediated largely by L-type calcium currents. At the molecular level SAN cells express both Ca_v_1.2 and Ca_v_1.3. Ca_v_1.2 is the classic L-type channel present throughout the heart that is responsible for calcium entry promoting calcium-induced calcium release from the sarcoplasmic reticulum and in the SA node is responsible for some of the action potential depolarization [7]. In contrast, Ca_v_1.3 activates at more hyperpolarised potentials and contributes to pacemaking [8,9]. Sodium currents are less obvious as the main driver of depolarization. However, tetrodotoxin-resistant and sensitive currents have been described and these may be present in the more peripheral parts of the node adjacent to the atrial tissue [5]. Sodium channel mutations in SCN5A result in cardiac conduction disease [10]. SCN10A has also been associated with conduction albeit in the atrioventricular node [11]. Repolarisation of the action potential is achieved by inactivation of L-type calcium currents and opening of a number of potassium channels. The latter are discussed in more detail below. The SAN action potential lacks a notch and plateau phase (phase 1 and 2) that is characteristic of the ventricular action potential.

**Figure 2.**
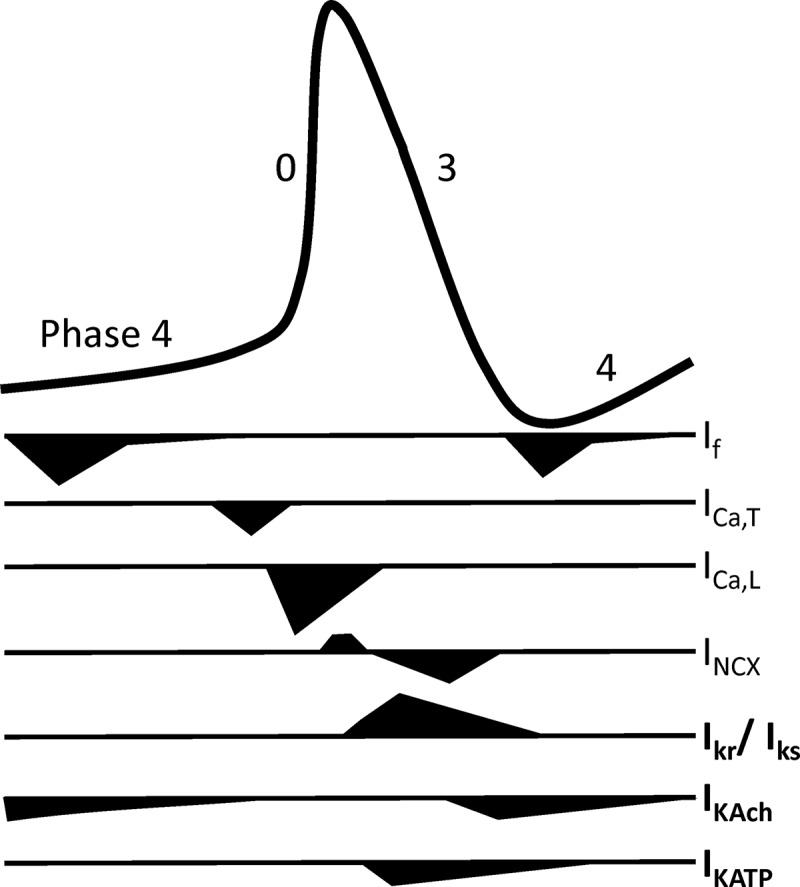
A cartoon showing the contribution of different currents during the SAN action potential and their contribution to the pacemaker depolarization and action potential repolarisation.

The second feature of SAN electrophysiology is the slow diastolic depolarization also known as the pacemaker potential. In the initial phase this is mediated by sodium influx via I_f_, largely constituted by the HCN4 subunit, and as depolarization occurs progressively by T-type calcium channels and finally L-type calcium channels, specifically Ca_v_1.3, just prior to threshold and initiation of phase 0. Ca_v_1.3 knockout mice have highly unstable pacemaker function with bradycardia and episodes of sinus pauses [12]. The major T-type subunit in the adult animal is Ca_v_3.1 and knockout mice are bradycardic [13]. This slow diastolic depolarization is opposed by inward rectifier potassium currents and other background currents (see below). Figure 2 summarizes the important currents underlying the SAN action potential.

## How is the rhythmicity of the diastolic depolarization generated?

One of the major recent advances has been an appreciation of the importance of intracellular calcium cycling in the genesis of the rhythmicity of the diastolic depolarization [4,14]. In addition to the membrane ion channels detailed above there is also expression of the sodium-calcium exchanger in the sarcolemma of the SAN myocytes [15,16]. The sodium-calcium exchanger transports three sodium ions for one calcium and thus has the potential to generate a net inward current when extruding calcium [17]. Calcium is released from the sarcoplasmic reticulum even during diastole when the cell is quiescent and in ventricular cells this is manifest as calcium sparks [18]. In SAN myocytes however these local calcium releases are larger involving a number of release sites [19–21]. In permeabilised cells the effects of membrane ion channels are removed and in these conditions these events are periodic in contrast to the stochastic nature of sparks in ventricular myocytes [22]. The SA node does not have t-tubules and calcium release is sub-sarcolemmal. The timing of the spontaneous calcium release is in the latter part of the diastolic depolarization and the activation of the inward sodium-calcium exchanger current is exponential ultimately leading to the threshold potential [15,21]. The SAN seems to have higher levels of the calcium pump, SERCA2, present in the membranes of the sarcoplasmic reticulum and in inducible knockout mice the heart rate is substantially slowed [23,24]. In addition, phospholamban, which in its unphosphorylated state inhibits SERCA2, is reduced in expression and basally phosphorylated compared to the ventricle [25]. Pacemaking and spontaneous calcium release are inhibited by cyclopenzoic acid and ryanodine and heart rate is slowed in a mouse with knockout of the sodium-calcium exchanger [16,19,20,26]. These observations all support the importance of a fundamental calcium clock in generating the rhythmic oscillator SAN discharge which integrates with the activity of membrane ion channels.

## Potassium channels and pacemaking

Given these features of SAN electrophysiology, the opening of potassium channels can contribute in two ways. In the first action potential repolarisation can be accelerated attenuating the contribution of the action potential to the cycle length and thus potentially increasing heart rate. In contrast an increase in potassium conductance during the slow diastolic depolarization will increase the maximum diastolic potential and slow the rate of pacemaker depolarization leading to a slower heart rate. The exact net contribution depends on the properties of the potassium current. If the current predominates around the resting membrane potential, such as with inwardly-rectifying potassium channels, then the net effect will be on the diastolic depolarization. In contrast if active at more depolarized potentials this could predominantly influence repolarisation. In principle both effects might occur and the net effect on heart rate would be difficult to predict. Modelling in these circumstances might be helpful in aiding the understanding of the physiological consequences of changes in a particular potassium conductance. The regulation of these currents could also be potentially important: increased potassium currents during the diastolic potential leading to heart rate slowing. It is also important that action potential duration adapts by shortening during high heart rates otherwise regular pacemaking would fail. Adrenergic modulation of repolarising potassium conductances is a mechanism to ensure that and this is discussed below.

## G-protein gated inwardly rectifying potassium (GIRK) currents and other background currents

All cardiac myocytes express a strong inwardly-rectifying potassium current. In ventricular cells this is known as I_K1_ and is accounted for by members of the Kir2.0 family of inward rectifiers [9,27,28]. In contrast in the SAN and atrial cells the current is less strongly inwardly-rectifying but is characteristically increased by muscarinic agonists such as acetylcholine and carbachol (“I_KACh_”) [29]. This current is a heteromultimer of Kir3.0 subunits that in cardiac cells is constituted of Kir3.1 and Kir3.4 with perhaps some homomultimers of Kir3.4 [30–32]. The differential expression is not absolute for example there are some data indicating expression of I_KACh_ in the ventricle [33].

Activation of I_KACh_ is characteristically inhibited by muscarinic antagonists and activation is inhibited by pertussis toxin implicating heterotrimeric G-proteins in the regulation [34]. After some controversy, it became clear that it was the G_βγ_ subunit, not the inhibitory Gα, that directly activated the channel complex and in many ways this is now the paradigmatic example of modulation of an effector by G_βγ_ [35,36].The domains on the Kir3.0 subunits that bind the G_βγ_ subunits and also the key residues on G_βγ_ have been mapped [36,37]. There are crystal structures of the channel complex with and without G_βγ_ subunits bound [38]. Anionic phospholipids particularly phosphatidylinositol- [4,5]-bisphosphate and sodium are known to be key modulators of the gating and a number of site-directed mutagenesis studies together with structural work have suggested models predicting how this might occur [38–40]. However the inhibitory G-protein α subunit also participates in determining the selectivity of activation but not directly in activation itself [41]. There are strands of evidence pointing to models in which the inhibitory heterotrimeric G-protein is complexed with the channel and receptor prior to activation [42–46].

The pathway delineated by genetic, physiological and pharmacological studies involved the activation of M_2_ muscarinic receptors and the dissociation of the inhibitory heterotrimer with the G_βγ_ activating GIRK channels in the SAN [47–50]. However the GTP bound inhibitory G_α_ subunit can also inhibit adenylate cyclase and reduce levels of cAMP reducing I_f_ [51]. Thus there are two possible mechanisms for the reduction of heart rate via the parasympathetic arm of the autonomic nervous system: one involving GIRK and another via I_f_. The relative importance of these has been debated over the years. Initially, low receptor occupancy was associated with I_f_ inhibition whilst higher receptor occupancy was needed for I_KACh_ activation [52]. The cloning of the Kir3.0 channel subunits enabled the development of mice with global genetic deletion of GIRK4 (kcnj5). These mice together with other experimental observations clearly implicate GIRK channels and I_KACh_ in heart rate regulation in physiological conditions [48,53].

GIRK channels are also important in the recovery of heart rate: GIRK4 knockout mice when exercised show a delay in the time taken to return to the resting heart rate [54]. This is intriguing as a delayed recovery of heart rate with exercise is associated with poor outcomes [55]. Regulator of G-protein signalling 6 (RGS6) is also critical to heart rate regulation [56,57] and acts as the predominant GTPase activating protein for inhibitory G-proteins in the SAN. The net result of this is to inhibit signalling but it also delays the recovery to the inactive state once the agonist is withdrawn. Intriguingly, in our recent genome wide association study in man examining the genetic architecture of heart rate response to exercise we implicated the genes encoding M_2_ muscarinic receptor, acetylcholinesterase and RGS6 in determining recovery responses after exercise [58]. Furthermore, genetic deletion or pharmacological inhibition of GIRK4 may also ameliorate the bradycardic and proarrhythmic effects of HCN4 and Ca_v_1.3 deletion [59,60].

## Repolarising currents

The major repolarising currents in ventricular myocytes are I_Kr_ and I_Ks_ and both components are also present in SAN myocytes [9,61]. I_Kr_ is named as such because it is rapidly activating and the currents are characteristically inwardly rectifying due to its unique inactivation properties [62]. I_Kr_ is composed of hERG (human ether-a-go-go related gene) and possibly a β subunit of the KCNE family though this is still controversial [63,64]. I_Kr_ is an important current in SAN cells [65,66]. For example, the inhibitor E-4031 leads to significant perturbation of the SAN action potential and dofetilide (another blocker) slows pacemaking [61]. In contrast, I_Ks_ is characteristically activates slowly and the channel complex is formed by KCNQ1 (Kv7.1) and the β subunit KCNE1 [67,68]. There may be some species differences in the relative magnitudes of I_Kr_ and I_Ks_ but I_Ks_ is also clearly present in the SAN [69,70]. One key property of these currents, in particular I_Ks_, is the augmentation by β adrenergic signalling: it is key in shortening the action potential duration during high heart rates [70,71]. Activation of β receptors leads to PKA stimulation and direct phosphorylation of residues S27 and S92 in the channel N-terminus [72]. The increase in current requires KCNQ1 to be associated with KCNE1and is dependent on a protein kinase A anchoring protein (AKAP) yotiao\AKAP9 [72,73]. Unlike I_Ks_ regulation, there is no agreement on whether I_Kr_ currents can be increased by β adrenoreceptor modulation [74,75]. Uniquely in the SAN, calcium-calmodulin kinase II may activate I_Ks_ and intriguingly this could affect calcium handling and channel modulation too [76].

Background potassium currents carried by twin pore channels are difficult to investigate given their poor pharmacology. However a recent study using cardiac-specific TREK1 knockout mice revealed that these mice were bradycardic and predisposed to sinus arrest [77]. Thus it seems likely that TREK1 also contributes to repolarisation.

## K_ATP_ channels underlie repolarising currents in the SA node

ATP-sensitive potassium channels are widely distributed in the heart. In general the focus has largely been on channels present in the ventricle but characteristic currents can also be recorded in the atria and in the conduction system including the SAN [78,79]. The defining property of these channels is their sensitivity to intracellular nucleotides and they are activated by declining ATP levels and\or increasing magnesium ADP levels. The channel complex is made up of four pore-forming inwardly-rectifying Kir6.0 subunits (Kir6.1, Kir6.2) together with four regulatory sulphonylurea receptors (SUR1, SUR2A, SUR2B) [80–82]. We now understand these channels well as molecular machines underpinned by exhaustive mutagenesis work and more recently by structural studies. Cryo-electron microscopy has shown how ATP binds to the pore-forming subunit and how MgADP interacts with the nucleotide binding domains in the sulphonylurea receptor [83–85]. Despite the initial description of these channels in the heart [86] there are still questions as to their exact physiological role. Kir6.2 is thought to underlie the classic current present in pancreatic β cells and ventricular cardiomyocytes [87]. Kir6.2 global knockout mice are unable to tolerate high intensity exercise partly due to impaired cardiac performance [88].

There has been comparatively less work on the Kir6.1 subunit though it has been known since it was first cloned that it is widely expressed [89]. Furthermore, there exists, particularly in smooth muscle, a K_ATP_ channel with a lower single-channel conductance (35 pS vs 70 pS) with an absolute dependence on cellular nucleotide diphosphates for channel opening. In some papers this led to the channel being called a “K_NDP_” [90]. In addition, ATP seems less potent in causing channel inhibition [90,91]. This current is recapitulated in heterologous expression systems by the co-expression of Kir6.1 and SUR2B [92] and smooth selective deletion of Kir6.1 (kcnj8) in mice abolished the current present in isolated single smooth muscle cells [93].

K_ATP_ currents are present in single isolated SAN cells and they were abolished in cells isolated from the Kir6.2 knockout mouse [94]. Interestingly single channel studies revealed a single channel conductance of ~50 pS in contrast to the situation in the ventricle where it is closer to 70–80 pS [95]. There are reasons to believe that pacemaker function may also be influenced by K_ATP_ channels containing the Kir6.1 subunit. Mice with global genetic deletion of Kir6.1 develop SAN failure and also heart block which leads to sudden death [93,96]. In our own studies we have studied mice with selective deletion of Kir6.1 in vascular smooth muscle or endothelium [93,97]. These mice do not show the rhythm disturbance even with provocations that might provoke vasospasm and suggest by exclusion a potential role for Kir6.1 in the SA and atrioventricular node [93,97].

We have explored this question in the mouse using cre\loxP technology [98]. We made use of a cre driver line that allows selective deletion of genes within the adult conduction system on the addition of tamoxifen [98,99]. The resulting knockout mice are labelled as cKO. We first isolated K_ATP_ currents in single SAN cells and find that only approximately half the cells contain currents [98]. This is not surprising given the heterogeneous electrophysiological nature of the SAN [100]. Importantly this number of cells was significantly reduced in cKO mice [98]. The pharmacology of the response is also interesting with diazoxide activating and tolbutamide inhibiting and this profile is more typical of SUR1 than SUR2 [101]. In murine atrial myocytes SUR1 underpins a significant population of K_ATP_ channels [102]. Furthermore when action potentials were measured in single SAN cells, there was delayed repolarisation in cKO cells resulting in prolonged cycle length [98].

The murine model also allows the investigation of the *in-vivo* physiological consequences of the Kir6.1 deletion [103]. Using implanted telemetry probes, we measured heart rate and ECG parameters in awake mice over a prolonged period. cKO mice are bradycardic and in some mice there were episodes of sinus arrest which were not seen in littermate controls [98]. Furthermore, there was an indication of atrioventricular nodal dysfunction with an increase of the PR interval on the ECG [98]. We also saw some modest pathological changes with fibrosis in the SA node in a few of the cKO mice [98]. The phenotype is not as pronounced as in the global Kir6.1 knockout animal but the impairment of vascular reactivity and lack of protective responses may accelerate pathological damage [93,98].

In summary, our recent studies show that K_ATP_ channels are constitutively active in SA nodal cells. The current seems to influence repolarisation predominantly and this results in an increased cycle length. In-vivo this leads to bradycardia but there was also evidence of sinus pauses, heart block and pathological changes in the SA node [98]. It is surprising that there are no effects on the maximum diastolic membrane potential. Kir6.1 is a member of the inwardly rectifying family of potassium channel however the currents are more outwardly rectifying [98]. Additionally, it is plausible that there may be some cyclical regulation of K_ATP_ channel activity during repolarisation perhaps by calcium and\or cAMP and protein kinase A to explain this paradox. Signalling via cAMP seems to be significantly adapted in the SA node. Adenylyl cyclase is constitutively active and this leads to basal protein kinase A activation [104]. The SA node in contrast to the ventricle expresses calcium sensitive adenylyl cyclase isoforms [105]. PKA activity, and also that of calcium calmodulin dependent kinase II, is necessary for normal pacemaking as inhibitors of these kinases lead to significant slowing of beating [25,106]. Given the sensitivity of both adenylyl cyclase and calcium calmodulin dependent protein kinase II to calcium there may also be cyclical variations in activity that, in addition to phosphorylating phospholamban, may also phosphorylate K_ATP_ channels. On the background of high phosphatase activity this may lead to variations in beat-to-beat channel activity and account for the prominence of currents during repolarisation. It is also known that K_ATP_ channels can be regulated by calcineurin [107,108]. It is known that Kir6.1-containing channels are prominently regulated by hormonal pathways and protein kinases both in heterologous and native tissues [93,109,110].

## The response of the SAN to hypoxia

The sinus node is prone to disease in particular sick sinus syndrome which leads to a pathologically slow heart rate and can be accompanied by sinus arrest leading to dizziness and loss of consciousness [111,112]. The disease increases in incidence with age and has been attributed to progressive cell loss and fibrosis [111,112]. In addition, atrial fibrillation can be accompanied by sinus node disease and “tachy-brady” syndromes are well described [113]. However it also clear that there are changes in the expression of relevant pacemaking genes in the SA node with age. For example HCN4 and SERCA2 decrease in expression and this may be responsible for the decline in intrinsic heart rate with age [14,113–116]. It may also be contributing to physiological and pathological adaptations too [117,118]. The SAN is supplied by SAN artery which usually arises from the right coronary artery in man though there are anatomical variations. The SA node responds to hypoxia and ischaemia with heart rate slowing and ultimately failure due to exit block from the SA node into the atrium [119]. In modelling work, a major component of the response to hypoxia is the opening of K_ATP_ channels [119]. Potential shortening of the action potential duration is offset by increases in calcium current but the membrane potential becomes hyperpolarised and the pacemaker depolarization is slowed. This results in bradycardia and ultimately SAN failure. Interestingly, these effects are exacerbated by concomitant increased vagal activity [119].

**Figure 3.**
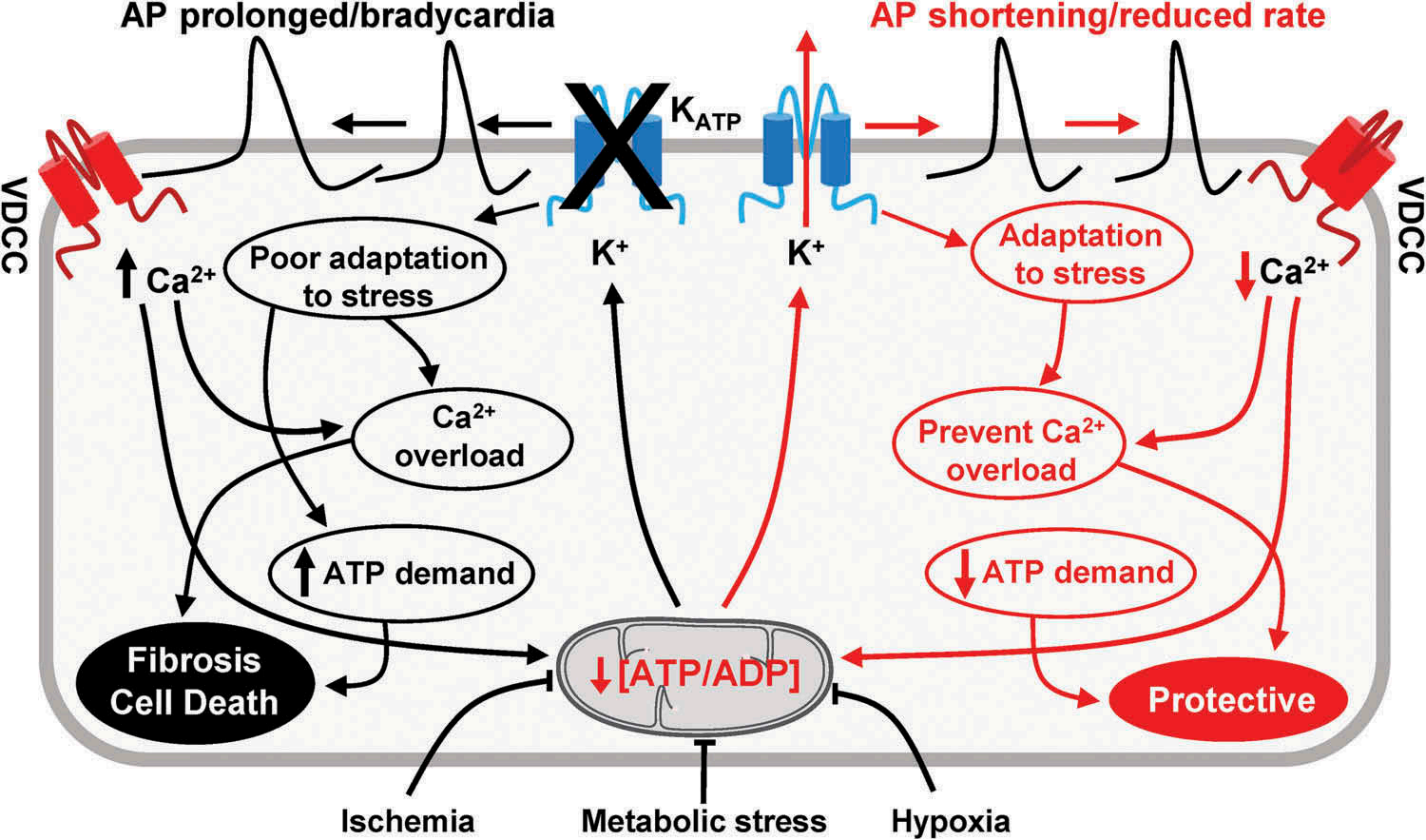
A cartoon showing the role of K_ATP_ channels in SAN function.

We investigated this issue in a different way [98]. Using an *ex-vivo* Langendorff preparation we measured spontaneous firing using a multielectrode array. We measured the sinus node recovery time by overdrive pacing the endogenous activity and then stopping the pacing and measured the time it takes for the SAN to recover spontaneous activity. This measure is used clinically to assess SAN function and in hypoxic conditions in control mice we found it became prolonged [98]. This may be a protective response where a reduction in activity helps preserve the viability of SAN myocytes. In contrast in the cKO mice the SAN recovery time did not prolong with hypoxia and under resting conditions was longer. This lack of flexibility may promote SAN damage. The contribution of K_ATP_ to SAN physiology and pathophysiology is summarized in Figure 3.

## Summary

Potassium channels play key roles in determining SAN repolarisation and the behaviour of the pacemaker potential. In this review we have placed in context the contribution of K_ATP_ currents to heart rate regulation revealed in our recent studies.
